# Consumer Decision-Making Based on Review Websites: Are There Differences Between Choosing a Hotel and Choosing a Physician?

**DOI:** 10.2196/jmir.5580

**Published:** 2016-06-16

**Authors:** Fabia Rothenfluh, Evi Germeni, Peter J Schulz

**Affiliations:** ^1^ Institute of Communication and Health Faculty of Communication Sciences Università della Svizzera italiana Lugano Switzerland

**Keywords:** physician rating website, qualitative research, health care quality assessment, electronic word of mouth, health care provider, physician choice, patient satisfaction

## Abstract

**Background:**

Web users are increasingly encouraged to rate and review consumer services (eg, hotels, restaurants) and, more recently, this is also the case for physicians and medical services. The resemblance in the setup and design of commercial rating websites (CRWs) and Web-based physician rating websites (PRWs) raises the question of whether choice-making processes based on the two types of websites could also be similar.

**Objective:**

This qualitative study sought to explore the extent to which consumer decision making based on Web-based reviews is the same for consumer services (ie, choice of a hotel) and health services (ie, choice of a pediatrician), while providing an in-depth understanding of potential differences or similarities.

**Methods:**

Between June and August 2015, we carried out a total of 22 qualitative interviews with young parents residing in the German-speaking part of Switzerland. Participants were invited to complete 2 choice tasks, which involved (1) choosing a hotel based on the commercial Web-based rating website TripAdvisor and (2) selecting a pediatrician based on the PRW Jameda. To better understand consumers’ thought processes, we instructed participants to “think aloud”, namely to verbalize their thinking while sorting through information and reaching decisions. Using a semistructured interview guide, we subsequently posed open-ended questions to allow them to elaborate more on factors influencing their decision making, level of confidence in their final choice, and perceived differences and similarities in their search for a hotel and a physician. All interviews were recorded, transcribed, and analyzed using an inductive thematic approach.

**Results:**

Participants spent on average 9:57 minutes (standard deviation=9:22, minimum=3:46, maximum=22:25) searching for a hotel and 6:17 minutes (standard deviation=4:47, minimum=00:38, maximum=19:25) searching for a pediatrician. Although the choice of a pediatrician was perceived as more important than the choice of a hotel, participants found choosing a physician much easier than selecting an appropriate accommodation. Four main themes emerged from the analysis of our interview data that can explain the differences in search time and choice confidence: (1) trial and error, (2) trust, (3) competence assessment, and (4) affect and likeability.

**Conclusions:**

Our results suggest that, despite congruent website designs, individuals only trust review information to choose a hotel, but refuse to fully rely on it for selecting a physician. The design and content of Web-based PRWs need to be adjusted to better address the differing information needs of health consumers.

## Introduction

Web-based review platforms allow consumers to post statements about products and services on the Internet by means of positive or negative reviews, also called electronic word of mouth [1]. Such websites are numerously accessible for purchases of physical goods and services (eg, amazon.com or zalando.de), as well as tourism and gastronomy (eg, booking.com or tripadvisor.com). Since the early 2000s, ratings have also been accessible for medical services, including the choice of physicians. Web-based physician rating websites (PRWs) “collect and present information about patients’ experience and satisfaction with individual physicians and their practices” [2].

Previous research has shown that the use of PRWs has increased over the last decade. In 2012, about 36% of Americans reported to have searched for a physician on the Internet, whereas 65% were aware of such ratings [3]. In European countries, such as Germany, in contrast, far fewer people have heard of (32%) or used (25%) such rating websites [4]. In Switzerland, awareness and use are estimated to be even lower as patients can presently post only positive reviews about their physician because of legal restrictions, which are supposed to be lifted in 2016 or 2017 [5]. Yet, PRWs’ impact on consumers’ final consultation decisions seems to be considerable: 65% of German PRW users have consulted a physician based on the ratings provided by these websites [6], and 30% of American consumers have checked PRWs before consulting a physician [7]. Younger generations, in particular, seem to be increasingly relying on the Internet when selecting a doctor. More than a quarter of young parents surveyed in the United States indicated that they had selected the pediatrician for their child on the Internet [8,9].

Past research has pointed out ethical issues related to the use of PRWs [10-13,19] has focused on the prevalence and content of PRWs [2,6,7,11,14,16-18,20] and has provided an initial understanding of individuals’ choice-making process when selecting a doctor on the Internet [8,15,43,44]. A study by Hanauer and colleagues [8] found a strong impact of physician ratings on patients’ final choice. In a Web-based experiment, 22% of parents who were prompted to find a new pediatrician for their child had followed the advice of their neighbors, whereas 46% did so when the neighbors’ recommendation was in line with positive online ratings of that physician. Nevertheless, only 3% consulted the pediatrician recommended by their neighbors if the doctor’s Web-based reviews were negative [8]. Furthermore, Grabner-Kräuter and Waiguny [15] found that the review style (factual or emotional) and the review number influenced individuals’ Web-based physician selection. Specifically, a high number of reviews resulted in a more positive attitude toward a physician, whereas the style mainly affected the reviewer’s perceived expertise. These results have shed new light on the influence PRWs can have on patients’ decision making. Yet, up to date, an in-depth understanding of how individuals choose a physician based on PRWs is missing. Similarly, the extent to which consumers’ decision-making approach could be similar as for commercial rating websites (CRWs) is largely unknown.

**Figure 1:**
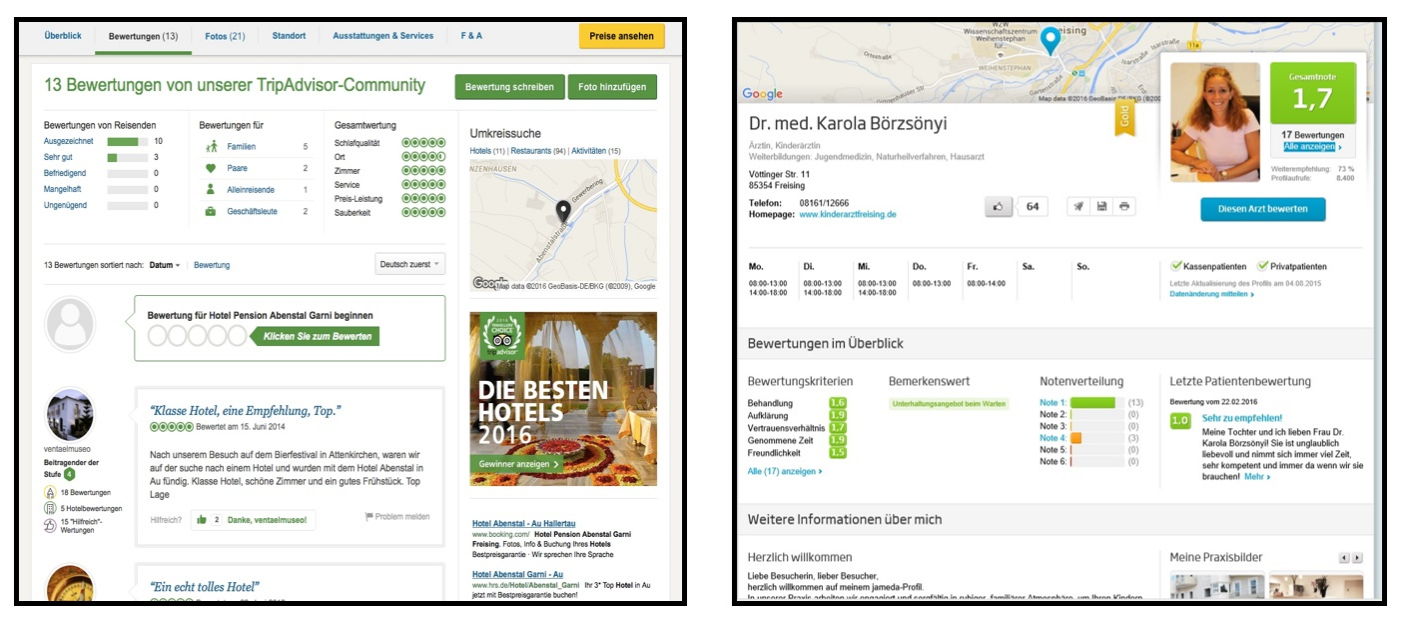
Screenshots of the hotel rating website tripadvisor.de [21] (left) and the physician rating website jameda.de [22] (right) retrieved on March 2, 2016.

The design and setup of CRWs holds significant similarities to that of PRWs (see Figure 1). Users can find general information, rate predefined aspects of products and services, write open-ended reviews, check the location and contact information, and see pictures. Due to the high visual similarity of PRWs and CRWs, we sought to investigate whether similarities could also exist with regard to the way that consumers reach their decisions based on these websites. Specifically, the objectives of the study were (1) to explore the extent to which the decision-making approach to selecting a physician is the same as for other consumer services or products (ie, choice of a hotel) and (2) to understand how potential differences in the search strategy can be explained.

### Theoretical Background

Extensive research has been conducted about the users, recipients, search, and decision-making processes on commercial Web-based ratings, reviews, and electronic word of mouth [23]. However, whether or to what extent this research can be translated to user behavior on PRWs (due to a similar setup and design of CRWs and PRWs) still needs to be investigated. How individuals make sense and understand their surroundings has theoretical roots in educational psychology. Resubsumption [24-27] and recategorization theory [27,28] outline how individuals reason when they encounter new phenomena, when they apply the same frame of logic that they learned from a similar earlier experience (monotonic processing) or a different one (nonmonotonic learning). Monotonic processes entail the learning of new information without any changes to the existing knowledge, whereas nonmonotonic learning coincides with changes in cognition, such as in attitudes, beliefs, conceptual or theory change, or deep learning [26,29]. Therefore, when individuals are faced with new situations or information, they by default anchor what they see, read, or perceive monotonically into a scheme that they are already familiar with [25]. In other words, they rely on resubsumption, which is “the process by which an existing theory is considered to be capable of encompassing and explaining additional experiences or phenomena and can occur before any confrontation with anomalies” [27]. In this study, we investigated whether the similar setup and design of CRWs and PRWs led to monotonous processes and subsequently analogous choice making despite the dissimilar attributes of the services that were to be selected (hotel vs physician).

## Methods

### Study Design and Participant Recruitment

A qualitative approach was employed to provide an in-depth understanding of consumer decision making. We used the consolidated criteria for reporting qualitative research (COREQ) to describe the study methods [30] (Multimedia Appendix 1). On approval of the study protocol by the Ethics Committee of the Università della Svizzera italiana (24.6.2015 CE 2015-5), we performed a total of 22 in-depth interviews with young parents residing in the German-speaking part of Switzerland. Main eligibility criteria included (1) age older than 18 years, (2) fluency in German, and (3) being a parent of a child aged younger than 3 years with no diagnosed physical or mental condition. We used a number of purposive sampling strategies to identify, notify, and invite participants who could cover a wide spectrum of perspectives and experiences: we distributed flyers at venues frequented by the target population (eg, child day care centers, parents’ associations) and liaised with midwives visiting young parents at their homes. Snowball techniques were also used, that is, initial participants were asked to pass on information about the study to other people in their networks. Parents interested in participating either gave permission to be contacted or directly got in touch with the research team (via phone) and arranged a convenient date and time for the interview. This multifaceted approach resulted in the recruitment of 22 young parents, out of whom 12 were females. Participant sociodemographic characteristics are presented in Table 1.

**Table 1:** Participant characteristics.

Variable	Category	n	%
Gender	Male	10	45.5
	Female	12	54.5
Age group	26-30 years	8	36.3
	31-35 years	10	45.5
	36-40 years	4	18.2
			
Education	University degree	4	18.2
	Applied science university degree	10	45.5
	Apprenticeship	8	36.3
			
Working status	Full time	9	40.9
	Part time	11	50.0
	Not working	2	9.1
			
Prior parenting experience^a^	No	15	68.2
	Yes	7	31.8
			
Self-reported IT proficiency^b^	High	14	63.6
	Medium	4	18.2
	Low	4	18.2
			

^a^Refers to being a first-time parent or being a parent of an older child at the time of the interview.

^b^As perceived and reported by study participants.

### Data Collection

Data for this study were collected from June to August 2015, through one-to-one, face-to-face interviews that lasted about 1 hour. The first author (FR), a social scientist with substantial training in qualitative research, conducted all interviews either in a private room at the University of Lucerne or at participants’ homes (based on their individual preferences). At the beginning of the process, the interviewer introduced herself, indicating that she is a PhD student focusing on topics related to how consumers reach their decisions based on Web-based reviews. She then invited informants to complete 2 choice tasks, which involved (1) choosing a hotel based on the commercial Web-based rating website TripAdvisor and (2) selecting a pediatrician based on the PRW, Jameda. To assimilate the search experience for all participants, they were instructed to search for a hotel and a pediatrician in the town of Freising (Germany), whereas the dates of the hotel stay were fixed for all interviewees and the budget restricted to CHF 50-100 per night (Textbox 1). The costs were automatically displayed in Swiss Francs as the page was accessed from Switzerland. In this way, the number of possible options for hotels and pediatricians was limited to 8 and 7, respectively. To better understand consumers’ thought processes, we invited participants to “think aloud” [31], namely to verbalize their thinking while sorting through information and reaching decisions. Using a semistructured interview guide, we subsequently posed open-ended questions to allow them to elaborate more on factors influencing their decision making, level of confidence in their final choice, and perceived differences and similarities in their search for a hotel and a physician. The interview guide was developed on the basis of existing literature and our own interest in the topic; yet, it was not systematically pilot tested. Interviews were held until data saturation was reached. All parents signed an informed consent form before their participation in the study. On completion of the interviews, participants received CHF 20 to compensate for their travel or babysitter expenses.

**Overview of choice tasks**

Choice task 1:

Imagine that you are going on a 5-day trip to Freising near Munich, Germany, with your family. You are leaving on November 2, 2015, and you are coming back on November 7, 2015. Please go on tripadvisor.de (the first tab in the browser window that is already open on the laptop) to choose a hotel that fits your needs. Please keep in mind that your budget per night is between 50-100 Francs, so please do not modify this criterion. You have about 15 minutes to complete this task.

Choice task 2:

Imagine that you or your partner had to change jobs and you had to move to Freising near Munich, Germany. Once you move there, your child has high fever and you urgently need to see a pediatrician. You have not met anyone yet in Freising, so you decide to consult the Internet. Please go to jameda.de (the second tab on your laptop browser window) to choose a pediatrician for your child. You have about 15 minutes to complete this task.

### Analysis

With participant permission, all interviews were recorded, using a digital voice recorder, and fully transcribed, along with field notes taken during and/or after the process. Data were subsequently anonymized and coded using the qualitative data analysis software Atlas-Ti. We did not use any pre-existing coding frame; rather, we opted for identifying themes linked to the data themselves. Using the approach by Braun and Clarke to thematic analysis [32], data analysis was a multistage, recursive process, which involved moving back and forth throughout the following phases: (1) familiarization with the data; (2) generating initial codes; (3) searching for themes; (4) reviewing themes; and (5) defining and naming themes [32]. Analysis was performed on the original Swiss German transcripts by FR, who is a native Swiss German speaker, with a subsample translated in English and being analyzed independently by EG to verify emergent themes.

## Results

**Figure 2:**
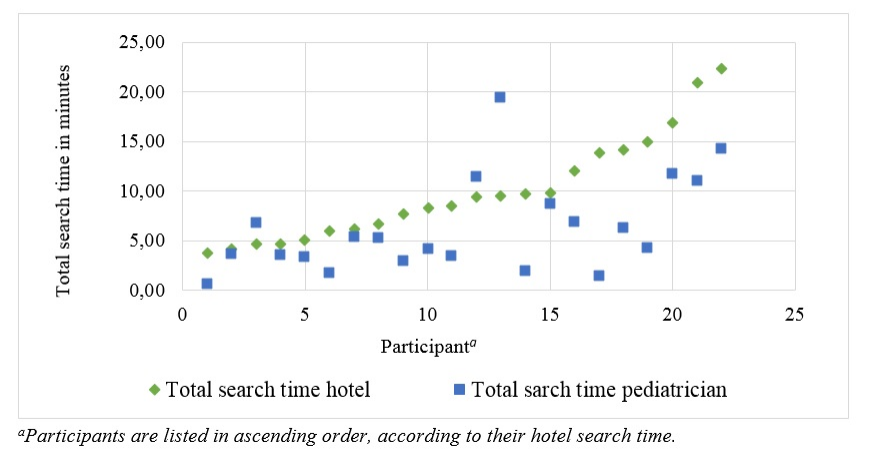
Total search time by task and participant.

Participants spent on average 9:57 minutes (standard deviation=9:22, minimum=3:46, maximum=22:25) searching for a hotel and 6:17 minutes (standard deviation=4:47, minimum=00:38, maximum=19:25) for a pediatrician. In fact, 19 of the 22 participants took longer to choose a hotel than a physician (see Figure 2). When choosing an accommodation, individuals stated to have specific criteria in mind that were based on their past experience with hotels and review websites. Therefore, parents strategically went searching for information about the location and accessibility of the hotel, the hygiene standards, the availability of children-friendly rooms and appliances, and the sleep quality. This took them considerable search time because finding such information was not always straightforward, especially if participants were not familiar with the TripAdvisor website.

By comparison, when choosing a pediatrician, individuals did not know exactly what to look for because all (except one) reported never having visited or heard of PRWs. Yet, due to the similar layout of the websites (Figure 1), almost all participants initially looked for the same type of information as they did when searching for a hotel. For example, individuals who had focused on pictures in their hotel selection on the CRW also checked out physicians’ photos first on the PRW. Likewise, participants who had been keen on reviews for hotels looked right away at comments when selecting a physician. One gender difference presented itself in the searches: although the large majority of men applied filters in their searches for both hotels and pediatricians (which allowed them to specify their criteria and cut down their choices), only a small minority of women did so. A male participant described the two search tasks:

It was very similar. Selecting what I am looking for, applying the filters accordingly, and then I started from the top. After that, I looked at the reviews and then some pictures. That’s important because these are the first things you look at. (…) It was quite similar.

BB, male, 34 years

Although the approach for choosing a hotel and a physician was highly similar at first sight, the interviews following the search tasks displayed major differences between the use of CRWs and PRWs. Four main themes that emerged from the analysis of our interview data can explain the differences between the two search tasks: (1) trial and error, (2) trust, (3) competence assessment, and (4) affect and likeability. The 4 themes were often brought up in an interwoven manner. Frequently one theme led to another in explaining the decision process and the reasons why physician choice was easier and carried more confidence than the hotel selection. Partial overlap of the themes was therefore inevitable.

### Theme 1: Trial and Error

In contrast to the choice of an appropriate accommodation, where individuals searched for detailed criteria that were important for them, parents unanimously applied a “trial and error” approach when choosing a pediatrician. Participants stated that, regardless of the kind or quality of information provided on PRWs, they would only know after the first visit whether they felt a connection with a physician or not. Hence, they would try a first consultation and subsequently decide to stay with that pediatrician or to switch to another doctor:

I basically trust all physicians and if I had visited a physician and somehow felt like “no, this wasn’t quite right,” then I would maybe search again.

AB, female, 30 years

I would go and give him a try and if it doesn’t work or doesn’t fit, I would change.

SKG, female, 34 years

Participants justified this “trial and error” approach by stating that the costs and stakes for choosing a pediatrician and a hotel were unequal. Although the choice of a physician was described as more important, the felt pressure to choose an appropriate accommodation seemed higher. Participants explained that while they had to spend their own money on accommodation, pediatrician visits were typically covered by the health insurance. This meant that the financial costs at stake were higher for the hotel selection, thus the choice of hotels had to be made more thoroughly. In addition, participants voiced profound trust in the medical profession, and hence, they perceived any of the listed doctors to be competent to treat their child (see theme 2). Therefore, individuals could afford to check out a pediatrician and then consult someone else if they were not satisfied. Furthermore, individuals were to invest scarce free time in an accommodation of their choice, whereas pediatricians visit would be of short duration and, therefore, would require a minor time commitment. Therefore, participants felt less pressured to right away make an optimal choice of pediatrician, which subsequently shortened their physician search effort and time compared with a hotel.

The hotel is more about something like holidays, something nice and the physician is a necessity. It isn’t somewhere/something where you go to relax. That’s why there is a difference. I think with a physician, your insurance actually pays most of it. And with the hotel you have to pay but you can also freely choose and that’s why you want a good value for your money.

AB, female, 30 years

The “trial and error” approach was also explained by the usage behavior of the different websites. PRWs could serve as a way to get an initial overview of the available doctors in an area, to make a first contact with a physician. The hotel rating website on the other hand was expected to provide a set of specific information that allows individuals to reach a final choice or to book an accommodation on the spot. Individuals compared the PRWs with an improved version of a phone book, in which they would otherwise look up the physicians’ contact information. Even though Web-based PRWs were considered as a better information source than a phone book, the additional information on the website was still perceived as insufficient to make a definite pediatrician choice. On the other hand, with the hotel choice, they felt confident to make a definite choice based on a rating website like TripAdvisor.

Yes, it is quite difficult but the website provides you at least with more information than the phone book, which would be the alternative. At least that’s how it (pediatrician choice based on the phone book) was done when there were no alternatives yet. In that sense, it is still a gut feeling decision in the end: I don’t know what will await me but I would just have to try once. For example with a physician, if I wasn’t satisfied, I could go to someone else the next time around.

SI, female, 33 years

The different website consumption was also based on the awareness that the choice of hotel would be definite and nonreversible on arrival, whereas a first visit at a pediatrician was not binding. Therefore, a trial and error approach seemed affordable with a physician but not with a hotel:

Such a physician rating website would probably be useful to make a first contact but after that it is obviously very much about the feeling you get, the appearance and impression once you get there. With a hotel, you book and then you say afterwards “okay, that was great” and you may go again some other time. There it is about the best offer at that moment. It isn’t really that relevant.

LS, female, 27 years

### Theme 2: Trust

Although most participants searched for technical skill information about the doctors, a high-level respect toward physicians, and, more broadly, trust in the health care system was evident in participants’ accounts. They tried to find descriptive information about the qualifications and certifications of doctors because the information seemed more legitimate to them. Hence, they did not question the information provided by the website provider or the physician himself. On the other hand, reviews and ratings by former patients were often perceived as strange or even unacceptable; participants could hardly believe nor approve that the skills of a physician would be evaluated by lay people. This suspicion toward physician ratings led some individuals to not even look at available reviews. This cut down their search time because subsequently they would choose a doctor based on sociodemographic indicators, location or decision shortcuts, such as to take the first physician on the list or to select the doctor closest to their home. One mother described:

This is just a bit strange to me, this whole thing about ratings! Especially rating a physician –to me, this is just suspicious! Yeah, I just still feel like: it is a physician! (…) Well, I don’t know, I would probably just take the one that is closest to my house.

AB, female, 30 years

In contrast to the choice of pediatrician, participants’ confidence in hotels was lower. Accommodations of the same star ratings (certificates by an external expert source) for example, were thought to differ tremendously in terms of quality. This, in turn, increased the time they spent searching for a hotel, while it decreased their choice confidence. Participants expressed their trust in physicians by referencing the well-known and rigorous educational requirements that all certified doctors in Switzerland and Germany have to fulfill. Parents concluded that all physicians needed to pass a critical threshold of competence because, otherwise, they would not have been certified as medical doctors. All listed doctors would therefore be capable and able to help their child. Hence, the difference in technical competence among pediatricians was perceived to be minor or nonsignificant, whereas the quality of hotels despite equal star ratings could differ tremendously.

I always think: they are physicians! And I actually always have a positive attitude anyways. (…) that’s why I am just glad if someone is there who can help if there is a need.

SA, female, 30 years

I just think: “a physician is a physician.” Somehow I am just… They know what they are doing!

AB, female, 30 years

### Theme 3: Competence Assessment

Although participants displayed confidence and experience in evaluating the quality of a hotel, they perceived themselves less competent in assessing the quality of a physician. Due to this perceived inability to assess the quality of a physician and the subsequent helplessness that they felt, the vast majority of parents opted to trust physicians by default. One mother explained:

I don’t understand any of it (the diagnosis and treatment prescription), so I trust in that, what he tells me and then I just take that (the medication) and I do what he tells me to do.

EA, female, 34 years

Obviously there are differences (among physicians), it always depends upon what your problem is. But in the end… Yeah well, you also don’t know which one (pediatrician) is better than the other ones. You never know!

EA, female, 34 years

The perceived incompetence of health consumers to correctly evaluate the skills and abilities of a physician was not only restricted to one’s own experience of a consultation with a particular physician but individuals also attributed it to former patients/reviewers who appraised the competence of a pediatrician on PRWs. As one mother states, PRW reviews should be taken with a grain of salt because according to her, a regular patient, a parent of a patient, or she herself would not be capable of assessing physician competence:

“A very good physician” – who am I to evaluate that?!I cannot assess the diagnosis, I am not a doctor! Under most circumstances I cannot tell whether it (the diagnosis and treatment) was good or not. Just based on gut feeling, this one (pediatrician) was likeable to me. But that doesn’t help me here… she (this physician) can be super nice and thoroughly answer to all questions, but in the end, she could be telling the biggest rubbish and prescribe a medicine that doesn’t fit the actual disease symptoms. I just cannot be sure.

SI, female, 33 years

Parents found that there are more criteria that they could consider in their choice of hotels than those in the choice of physicians. This lack of knowledge about what makes a good physician and the resulting trust when realizing that one is not able to differentiate was not mirrored in the choice of hotels. Participants voiced confidence in their hotel assessment expertise. This is the reason why individuals’ search for an accommodation was more detailed, more structured, and focused:

A hotel rating website probably provides you with more criteria to choose from, which makes the decision more difficult. And here (referring to the PRW), I mean you have seven pediatricians. They are all somewhat quite similar, grades between 1 and 2.3 and I mean, there isn’t any one that is somehow very, very bad.

FG, male, 30 years

The general skepticism toward reviewers (as displayed in the citations previously) was dominant for reviews on PRWs. On the other hand, reviews by former hotel guests were given more weight and considered in more depth, whereas the reviews of physicians were perceived to be more situation dependent and therefore most likely less representative than for hotels. By stating this, individuals implied that situation-dependent reviews are not an indicator of the actual and true qualities of a physician. Reviews may be biased due to the reviewer’s personal involvement, and therefore, a trial and error approach would be necessary to create a personal opinion. One mother described this difference:

I find it difficult to give a grade to a doctor. For a hotel room it is much easier than for a physician because how you experience a physician is extremely dependent on the time, the day, and many other things. Yes. I think if you get unlucky, yeah… And many positive ones don’t even write reviews. But if you are angry, then you go onto the website and you say: I want to hurt this person with a bad review, yeah.

SA, female, 30 years

### Theme 4: Affect and Likeability

Informants often let the emotions that appraisals or pictures of the physician or hotel evoked guide their choice making. Both positive affect to get a good sensation about a choice option and negative affect that led individuals to turn down or avoid an option were present. However, participants stated that the choice of a pediatrician was much more emotion guided than the selection of a hotel. Interpersonal connection, gut feeling, and likeability played a large role in the choice of a doctor, whereas the commercial focus on price and offered facilities was crucial for the choice of hotels. As one father, who was in favor of reviews, described:

With a hotel it was much more important how much it costs and this component isn’t present for a physician because it will most likely be paid by the insurance. But other than that, they are very similar, especially at the beginning. Afterwards, I think the hotel is more about commercial aspects, while the physician is certainly more about feelings and that’s why the reviews from other people are much more important.

LS, male, 38 years

The likeability and connection with a physician was also described to be very subjective and highly dependent on the individual. As personal affect and likeability play an important role in the choice of a physician, participants would often admit to apply a trial and error approach based on their own experience because reviews or word of mouth did not seem representative:

One may say “he is an excellent doctor, you have to go to him!” and someone else says “oh no, I don’t agree at all”. This is the same for a midwife and for a dentist. You have to see it for yourself, if it fits for you and if you like it.

EA, female, 34 years

The first positive affect-evoking aspect was the perception of likeability of the physician. A variety of indicators were used, in particular, reviewers’ evaluations of personality aspects and sociodemographic information. The former included criteria, such as a physician’s sense of humor, the likeability, and the perceived child friendliness:

I find likeability, charisma, how he treats us, how he treats our child, how he talks to us, very important.

FG, male, 31 years

Sociodemographic factors typically consisted of the pediatrician’s age, the gender, or the perception that the pediatrician may share common interests with the parent. In addition, the cultural background was inferred from the pediatrician’s name, with doctors with German-sounding last names being preferred. Participants stated that knowing about a physician as a person gave them a positive gut feeling and that shared characteristics made them feel more confident about the first encounter:

I think physicians don’t have to hide behind these reviews and it would have just been nice if there would have been some more information about them or a picture of the practice maybe. That way you can already get a good feeling about where you are going for the visit and some trust is already built through the Internet I would say.

AW, female, 29 years

Furthermore, pictures were often used to make an inference about a person’s likability:

The picture is the most important indicator for me: is this person likeable or not? I am someone who trusts in the first impression. Obviously there is a possibility to convince me of the opposite but I can’t deny that if someone is likeable at first sight, then I can just go much more relaxed and comfortable to that visit than if someone isn’t.

RS, male, 32 years

In addition to aspects concerning the character of a physician, individuals were also interested in the beliefs that a pediatrician held. In particular, the attitude toward vaccination and alternative medicine posed key decision factors. Especially parents who valued alternative medicine and had a critical attitude toward vaccination tried to find information about pediatricians’ views on those topics. Once they found a pediatrician who shared their beliefs, it had a large impact on their decision as it increased confidence and reduced search time:

(...) if you don’ want to get your child vaccinated, then you have to really think hard which physician you want to consult, so that you can enforce that once you get there.

PS, male, 33 years

Although individuals tried to find a physician who evoked positive emotions, certain factors led them to turn away immediately. For hotel choice, negative affect was caused by pictures, a lack of child-friendly appliances or furniture, or information about the lack of hygiene. For physician choice, on the other hand, insufficient office organization or planning capacity and a lacking of willingness to communicate and inform parents about the treatment caused the arousal of negative emotion. When looking at reviews, physicians were judged in a milder way than hotels because the reviews were perceived to be more subjective and therefore of relative importance. Hence, individuals were less concerned about negative physician reviews, whereas negative comments about hotels were given much more weight:

A very nice doctor” and the next person states that he was in a bad mood. For me, these are not hard facts like you can find them for a hotel. With a hotel it certainly is subjective too but still, it is not quite the same for me.

SI, female, 33 years

That’s with every physician - they are also people and everyone has a bad day sometimes and most of the time it’s exactly then that people spread the word and harp on about it.

SA, female, 30 years

## Discussion

Our results offer insights into consumer decision making based on Web-based reviews. At first sight, participants in this study seemed to apply the same search strategy and decision mechanisms when choosing an accommodation and when selecting a pediatrician; on both websites, each individual focused on the same type of information to reach a decision, such as pictures of the rooms or the practice, reviews, or descriptive information about facilities and services or treatments offered. The highly similar layout of the two websites led them to start out in the same manner in the hotel search as in the physician selection. Yet, as the search proceeded, individuals became more skeptical and changed their approach through the discovery of differences between the choice of an accommodation and the choice of a physician. In the interviews after the choice tasks, participants’ conceptualization of the two websites appeared entirely different. The themes that emerged from our interviews describe this sense-making process or, consistent with resubsumption [24,25] and recategorization theory [27,29], the change from monotonic processing (both websites are the same and should be approached equally) to nonmonotonic change (the differences between the 2 choices outweigh the similarities and therefore the decision-making process for the 2 choice changes).

Overall, our results suggest that, although the choice of a pediatrician was perceived as more important than the choice of a hotel, participants found choosing a physician much easier than selecting a suitable accommodation. Moreover, although participating young parents seemed to believe that their choice of hotel could have been ameliorated, they were more satisfied with their choice of pediatrician, despite the shorter search time. On the basis of our interview data, these discrepancies can be explained by the following factors: (1) the application of a “trial and error” approach in the selection of an appropriate medical doctor; (2) their high trust in medical expertise and the Swiss health care system; (3) participants’ perceived inability to properly evaluate the skills and abilities of physicians; and (4) the role of “gut feeling” and likeability (vs the use of explicit criteria) in choosing a physician.

A starting point to understand our findings lies in the economics literature, which has developed a typology of service goods, categorized according to the information asymmetry between provider/seller and customer/consumer [33,34]. The first type consists of the so-called “search goods.” They can be compared before purchase in terms of price and quality based on openly available information, which in turn leads to high levels of choice confidence [35]. Research has found that for search goods, the risk perception is lowest, whereas the search time (due to the strategic comparison of the goods) is highest unless the cost of searching would outweigh the price of the good [35]. For the second type, namely “experience goods,” information inequality is larger. Consumers are unable to assess the quality of this kind of product or service until tried, consumed, or experienced [34]. Therefore, search time is shorter until a decision is reached because a trial of the product is essential to evaluate the quality of experience goods [35]. For the third type, that is, “credence goods,” the information inequality between provider and consumer is the highest. Because individuals cannot assess the quality of the product even after consumption, the search time is lower and individuals rely more on interpersonal recommendations than public noncustomized information before purchase or selection [35].

Individuals’ description of the search process for an accommodation and a pediatrician was reflected in the categorization of the goods and services categorization above. Although the selection of hotels mainly carried attributes of an experience good, the choice of a physician could predominantly be classified as credence good. As participants in this study described, they were highly motivated to compare possible options for accommodation, as they would have to invest both their own money and their holiday time. In addition, they felt confident that, based on the information provided, they could choose a good hotel. The vast majority of participants also appeared to trust in the information provided on TripAdvisor and meticulously compared the available hotel choices. Yet, informants stated that they could only know if they would be satisfied with their choice once they experienced a stay at the chosen accommodation.

For the choice of the physician, on the other hand, individuals decided faster and described that they applied a trial and error approach. Participants often perceived that using a PRW would only marginally aid their choice and they preferred making an appointment and meeting the physician in person. Participants were sure that after a first visit, they would be able to understand if they had a good interpersonal connection with the doctor. However, they were aware that likability was not an indication of the quality of the physician or the correctness of the diagnosis and treatment they received. Therefore, individuals’ description of the Web-based physician choice resembled the attributes of a credence service; evaluation of the quality of treatment could almost never be assessed even after an appointment, neither by themselves nor by other laypeople who posted comments or reviews on PRWs. Hence, many participants were skeptical about comparing physicians, as previous research had suggested [36].

Despite the congruent findings between this study and existing literature on search, experience, and credence goods, there are some theoretical expansions that the results of this study suggest. First, the theory on good classification according to Nelson [33] poses that the trust into one’s own choice should be highest for search goods and diminish to be the lowest for credence goods. Nevertheless, our study suggests that in the context of health care, this may not hold true. Due to the high trust in physicians that individuals of this study held, the perceived riskiness of a credence good and the confidence in one’s choice is dependent on the context. Not only the attributes of the good or service but also an individual’s past experience, and the level of trust in the system in which that good is provided, should be taken into consideration. Trust may be a moderator or mediator in the search and choice making of credence good.

### Limitations

This study has certain limitations that need to be addressed. First, our results should be interpreted with caution, given that participants’ accounts were triggered by 2 hypothetical scenarios and not real-life occurrences (eg, an actual move of the family to a foreign place or a real illness of the child). Yet, we opted for using real websites in an effort to create a more realistic sense and enable informants to reflect on their prior experiences. Nevertheless, this also meant that we were not able to control for any modifications caused by the dynamic and volatile nature of these websites. In fact, the information that individuals viewed in the 2 choice tasks slightly differed from the start of the data collection (June 2015) toward the end (August 2015) because of newly posted comments and reviews. In order, however, to maximize the information that all participants would see and could base their choice on, price category for the hotel and visiting dates were fixed.

Second, the study sample came from the German-speaking part of Switzerland, in which education levels, health literacy, and numeracy have been found to be very high [37]. Previous research has shown that active search of physicians and correct evaluation of quality of care information to select a physician depended on these 2 characteristics [38-40]. Therefore, although our findings could be transferable to other contexts characterized by high health literacy and numeracy levels, they might be less relevant for contexts where these attributes are less pronounced.

Third, given the context-bound nature of qualitative research, our results should be interpreted in relation to the specific legal and factual circumstances of Switzerland. Specifically, Switzerland has not yet established a common electronic health record, which is state of the art for many other European countries. Apart from this, the legal situation for Swiss PRWs differs from its European neighbors. Calling on data protection laws, the Swiss physicians’ association (FMH) requested a ban of negative reviews for physicians practicing in Switzerland [5]. This has affected the dissemination and use of PRWs in the country, which can explain the low awareness of PRWs among study participants.

### Conclusions

Individuals’ page navigation on PRWs and CRWs appeared similar. However, their perception of the 2 choice tasks was different. Participants displayed greater confidence in their physician selection, but took less time to make that choice. Individuals were incapable to assess whether a physician was good or not, even if they reviewed available information on the review website. This led them to a “trial and error” approach; only after a first encounter, they would decide whether the doctor was a match in terms of likeability, yet they would still remain unsure about his or her technical qualities. On the other hand, for the hotel choice, participants were confident that they could obtain all necessary information on the Internet to choose a good hotel for themselves. The fact that they invested their own money and precious free time made them feel more responsible to make a good hotel choice. Therefore, the information provided on CRWs seemed to fit customers’ needs, whereas the similarly designed PRWs did not satisfy study participants due to the attributes of the 2 services in the choice tasks.

### Implications for Future Research

In this study, participants took less time to search for physicians than hotels, voiced a profound trust in the health care system, and applied a “trial and error” approach to choose a doctor. Previous research has found that particularly in developed health care systems, such as Switzerland or Germany, patients refrain from actively searching and comparing physicians [41]. Therefore, a replication of this study in a country where trust in the health care system is lower may bring additional valuable insights. PRWs and CRWs look very similar: both offer pictures, background information (location, contact information, and descriptions of the facilities and services offered), as well as numeric written reviews by former guests or patients. However, as this study showed, the 2 service goods cannot be treated equally because of their unequal attributes. This has implications for the information needs consumers or patients have and, subsequently, for the design and creation of PRWs. Although individuals seem to want mass media information sources for search goods, a combination of mass media and interpersonal information for experience goods, they rely heavily on their social network to obtain information for credence good choices [35]. Currently, PRWs are set up in the same manner as an experience good by combining mass media information (general information about the location, accessibility, qualifications, and so forth) of the physician with impersonal recommendations (such as anonymous reviews by former patients). Interpersonal information that is first of all trustworthy, and second, adjusted to individuals’ needs [35,42] is therefore required for credence goods. Hence, this study provides first indications to change the way PRWs are currently designed. Translating these requirements into a website would be a task for today’s Web designers and researchers to take on. In addition, comparisons between the choice making process for a physician and other experience and credence goods/services (such as for a plumber, hairdresser, or car mechanic) could provide further insights into users’ product or service-based information needs. The results of this study could serve as the basis for future research that focuses on individuals’ information needs when selecting a doctor on the Internet. Experimental tests could subsequently improve the design and, therefore, the use and navigation of Web-based PRWs.

## Appendix

Multimedia Appendix 1 COREQ checklist.

## Abbreviations

CRW: commercial rating website
PRW: physician rating website
