# Supplementary material for: Consumer Decision-Making Based on Review Websites: Are There Differences Between Choosing a Hotel and Choosing a Physician?
Source: J Med Internet Res. 2016 Jun 16;18(6):e129. doi: 10.2196/jmir.5580 (PMC4929347; doi:10.2196/jmir.5580)
Supplement: Multimedia Appendix 1 [file jmir_v18i6e129_app1.pdf]

Consolidated criteria for reporting qualitative studies (COREQ)

No. Item	Guide questions/description	Reported on Page #
**Domain 1: Research team and reflexivity**		
Personal Characteristics		
1. Interviewer/facilitator	Which author/s conducted the interview or focus group?	5
2. Credentials	What were the researcher's credentials? E.g. PhD, MD	5
3. Occupation	What was their occupation at the time of the study?	5
4. Gender	Was the researcher male or female?	5
5. Experience and training	What experience or training did the researcher have?	5
Relationship with participants		
6. Relationship established	Was a relationship established prior to study commencement?	N/A
7. Participant knowledge of the interviewer	What did the participants know about the researcher? e.g. personal goals, reasons for doing the research	5
8. Interviewer characteristics	What characteristics were reported about the interviewer/facilitator? e.g. Bias, assumptions, reasons and interests in the research topic	5
**Domain 2: study design**		
Theoretical framework		
9. Methodological orientation and Theory	What methodological orientation was stated to underpin the study? e.g. grounded theory, discourse analysis, ethnography, phenomenology, content analysis	6
Participant selection		
10. Sampling	How were participants selected? e.g. purposive, convenience, consecutive, snowball	4
11. Method of approach	How were participants approached? e.g. face-to-face, telephone, mail, email	4
12. Sample size	How many participants were in the study?	4
13. Non-participation	How many people refused to participate or dropped out? Reasons?	N/A
Setting		
14. Setting of data collection	Where was the data collected? e.g. home, clinic, workplace	5
15. Presence of non-participants	Was anyone else present besides the participants and researchers?	5

16. Description of sample	What are the important characteristics of the sample? e.g. demographic data, date	4
Data collection		
17. Interview guide	Were questions, prompts, guides provided by the authors? Was it pilot tested?	5
18. Repeat interviews	Were repeat interviews carried out? If yes, how many?	N/A
19. Audio/visual recording	Did the research use audio or visual recording to collect the data?	6
20. Field notes	Were field notes made during and/or after the interview or focus group?	6
21. Duration	What was the duration of the interviews or focus group?	5
22. Data saturation	Was data saturation discussed?	5
23. Transcripts returned	Were transcripts returned to participants for comment and/or correction?	N/A
**Domain 3: analysis and findings**		
Data analysis		
24. Number of data coders	How many data coders coded the data?	6
25. Description of the coding tree	Did authors provide a description of the coding tree?	N/A
26. Derivation of themes	Were themes identified in advance or derived from the data?	6
27. Software	What software, if applicable, was used to manage the data?	6
28. Participant checking	Did participants provide feedback on the findings?	N/A
Reporting		
29. Quotations presented	Were participant quotations presented to illustrate the themes/findings? Was each quotation identified? e.g. participant number	7-12
30. Data and findings consistent	Was there consistency between the data presented and the findings?	7-12
31. Clarity of major themes	Were major themes clearly presented in the findings?	7-12
32. Clarity of minor themes	Is there a description of diverse cases or discussion of minor themes?	7-12
